# Clinical and analytical comparison of six Simoa assays for plasma P-tau isoforms P-tau181, P-tau217, and P-tau231

**DOI:** 10.1186/s13195-021-00939-9

**Published:** 2021-12-04

**Authors:** Sherif Bayoumy, Inge M. W. Verberk, Ben den Dulk, Zulaiga Hussainali, Marissa Zwan, Wiesje M. van der Flier, Nicholas J. Ashton, Henrik Zetterberg, Kaj Blennow, Jeroen Vanbrabant, Erik Stoops, Eugeen Vanmechelen, Jeffrey L. Dage, Charlotte E. Teunissen

**Affiliations:** 1 Neurochemistry Laboratory, Department of Clinical Chemistry, Amsterdam Neuroscience, Vrije Universiteit Amsterdam, Amsterdam UMC, Boelelaan 1117, Amsterdam, 1081 HV The Netherlands; 2 Alzheimer Center, Department of Neurology, Vrije Universiteit Amsterdam, Amsterdam UMC, Amsterdam, The Netherlands; 3 Department of Epidemiology and Data Science, Vrije Universiteit Amsterdam, Amsterdam UMC, Amsterdam, The Netherlands; 4 Department of Psychiatry and Neurochemistry, Institute of Neuroscience and Physiology, The Sahlgrenska Academy at the University of Gothenburg, Mölndal, Sweden; 5 Wallenberg Centre for Molecular and Translational Medicine, University of Gothenburg, Gothenburg, Sweden; 6 King’s College London, Institute of Psychiatry, Psychology and Neuroscience, Maurice Wohl Institute Clinical Neuroscience Institute, London, UK; 7 NIHR Biomedical Research Centre for Mental Health and Biomedical Research Unit for Dementia at South London and Maudsley NHS Foundation, London, UK; 8 Clinical Neurochemistry Laboratory, Sahlgrenska University Hospital, Mölndal, Sweden; 9 Department of Neurodegenerative Disease, UCL Institute of Neurology, Queen Square, London, UK; 10 UK Dementia Research Institute at UCL, London, UK; 11 Hong Kong Center for Neurodegenerative Diseases, Hong Kong, China; 12 ADx NeuroSciences NV, Technologiepark 94, Gent, Belgium; 13 Eli Lilly and Company, Indianapolis, IN 46285 USA; 14 Stark Neuroscience Research Institute, Indiana University School of Medicine, Indianapolis, IN 46202 USA

**Keywords:** Alzheimer’s disease, Blood biomarkers, P-tau181, P-tau217, P-tau231, Phosphorylated tau proteins, Simoa, Ultra-sensitive immunoassays, Analytical validation, Clinical validation

## Abstract

**Introduction:**

Studies using different assays and technologies showed highly promising diagnostic value of plasma phosphorylated (P-)tau levels for Alzheimer’s disease (AD). We aimed to compare six P-tau Simoa assays, including three P-tau181 (Eli Lilly, ADx, Quanterix), one P-tau217 (Eli Lilly), and two P-tau231 (ADx, Gothenburg).

**Methods:**

We studied the analytical (sensitivity, precision, parallelism, dilution linearity, and recovery) and clinical (40 AD dementia patients, age 66±8years, 50%F; 40 age- and sex-matched controls) performance of the assays.

**Results:**

All assays showed robust analytical performance, and particularly P-tau217 Eli Lilly; P-tau231 Gothenburg and all P-tau181 assays showed robust clinical performance to differentiate AD from controls, with AUCs 0.936–0.995 (P-tau231 ADx: AUC = 0.719). Results obtained with all P-tau181 assays, P-tau217 Eli Lilly assay, and P-tau231 Gothenburg assay strongly correlated (Spearman’s rho > 0.86), while correlations with P-tau231 ADx results were moderate (rho < 0.65).

**Discussion:**

P-tau isoforms can be measured robustly by several novel high-sensitive Simoa assays.

**Supplementary Information:**

The online version contains supplementary material available at 10.1186/s13195-021-00939-9.

## Background

Alzheimer’s disease (AD) neuropathology is defined by amyloid-β (Aβ) accumulation in extracellular plaques and hyper-phosphorylated tau (P-tau) accumulation in neurofibrillary tangles [1–3]. Aβ and tau pathology can be visualized on positron emission tomography (PET) or in the cerebrospinal fluid (CSF) to assist AD diagnosis [1]. However, these methods are invasive, expensive, and not widely available in care settings, which hinder implementation and use. Therefore, there is a strong interest to develop blood-based biomarkers for AD.

Different studies using different assays and technologies showed that plasma P-tau isoforms, such as P-tau181, P-tau217, and P-tau231 are highly accurate and specific for detection of PET-confirmed Aβ and tau pathology across the clinical AD continuum [4–12]. Furthermore, plasma P-tau181, P-tau217, and P-tau231 have been reported to be already increased in Aβ-PET-positive but still tau-PET-negative individuals [5, 7, 11], suggesting sensitivity for early Alzheimer’s pathology. Moreover, plasma P-tau has strong value in differential diagnosis, with high accuracy for discriminating patients with AD from frontotemporal lobar degeneration [4, 5, 8, 11–14]. In addition, among patients with dementia with Lewy bodies, plasma P-tau identifies amyloid co-pathology [14, 15].

There is a debate whether specific P-tau isoforms have favorable accuracies for diagnosis of AD dementia. For example, some studies in CSF showed higher accuracy of P-tau217 than P-tau181 in CSF to detect AD pathological changes [16, 17]. Likewise, in a comparative plasma study, it was suggested that plasma P-tau217 might be a better biomarker as compared to P-tau181 [8], though this was not observed by an independent study with an updated form of this P-tau181 assay [18]. Plasma P-tau231 on the other hand was suggested to have greatest potential to detect AD pathology in the earliest disease stages [11]. The different plasma P-tau assays that are currently available employ different platforms and different antibody pairs for detection of the different isoforms. It is not yet known whether there are technical reasons, such as differences in binding affinities and specificity of antibodies or sensitivity of the platforms or biological reasons that explain potential differences in clinical performance between the P-tau isoforms.

Here, we present a head-to-head analytical and clinical comparison of six novel P-tau assays that were developed on the high-sensitivity Simoa platform, including three different P-tau181 assays (Eli Lilly, ADx NeuroSciences, and Quanterix), one P-tau217 assay (Eli Lilly), and two P-tau231 assays (ADx NeuroSciences, Gothenburg).

## Methods

### Plasma samples

For the analytical validation, left-over K_2_EDTA plasma of routine diagnostic measurements at the Clinical Chemistry department was used. No clinical data were collected. The plasma tubes were centrifuged at 1800x*g* for 10 min. To reach a high volume, the plasma of different tubes derived from different individuals was pooled. The different plasma pools were aliquoted into polypropylene tubes (Sarstedt, Germany) and stored at −80°C until use.

For the clinical validation, we selected 40 participants with AD dementia from the Amsterdam Dementia Cohort [19, 20] (average±standard deviation (SD) age 66±8 years, *n* = 20 (50%) female), who were diagnosed according the NIA-AA diagnostic AD criteria [21] and had a CSF biomarker-confirmed AD diagnosis. We selected 40 age- and sex-matched (age 66±8 years, *n* = 20 (50%) female) cognitively healthy control participants from the Dutch Brain Research Registry (Hersenonderzoek.nl; CSF AD biomarker status unknown) [22]. The minimal sample size required was calculated based on three different studies, one study for each of the three P-tau forms that were measured in our study (P-tau181, P-tau217, P-tau231) [4, 11, 23]. The power calculation showed that a study with a minimum sample size of 4–8 participants per group would be sufficient to achieve a power (β) of 80% and a level of significance (*α*, two sided) of 5% for detecting true differences in values between the patients with AD and the controls. K_2_EDTA plasma samples were obtained from all participants through venipuncture. After a 10-min centrifugation at 1800x*g*, plasma was aliquoted into 0.5-mL portions in polypropylene storage tubes (Sarstedt, Germany) and stored at −80°C until use.

Prior to the P-tau analyses, plasma samples were shortly thawed, vortexed, and centrifuged at 10,000x*g* for 10 min. All analyses were performed on the Simoa HD-X platform (Quanterix, Billerica, MA, USA) according to manufacturer’s instructions. All measurements for analytical and clinical validation of the P-tau assays were performed at the Neurochemistry Laboratory, Amsterdam UMC, VUmc, Amsterdam, the Netherlands, except for the P-tau231 Gothenburg assay, for which the measurements were performed at the Clinical Neurochemistry Laboratory, Sahlgrenska University Hospital, Mölndal, Sweden. All P-tau analyses were performed in duplicates.

### P-tau assays

The study included three P-tau181 assays (Eli Lilly, ADx, and Quanterix), one P-tau217 assay (Eli Lilly), and two P-tau231 assays (ADx, Gothenburg). The P-tau181 assay of Quanterix is commercially available (#103714, Simoa® P-tau-181 V2 Advantage Kit), which is a modification of an earlier published set-up using the same antibodies and calibrator [5]. Both Eli Lilly assays are prototype Simoa assays based on the earlier published Meso Scale Discovery (MSD) assay set-up [18]. Both ADx assays are new prototype assays including in-house developed antibodies. The P-tau231 Gothenburg assay is a prototype assay developed by the Neurochemistry Laboratory of the University of Gothenburg [11]. Assay characteristics are summarized in Table 1.

**Table 1:** Analytical characteristics of the six P-tau assays

		P-tau181		P-tau181		P-tau181		P-tau217		P-tau231		P-tau231	
**Assay characteristics**													
*Assay*	*Provider*	**Eli Lilly**		**ADx NeuroSciences**		**Quanterix**		**Eli Lilly**		**ADx NeuroSciences**		**Gothenburg (NA)**	
	*Status*	Prototype		Prototype		Commercial		Prototype		Prototype		Prototype	
	*Catalogue number*	N/A		N/A		103714		N/A		N/A		N/A	
	*Biofluid*	EDTA plasma		EDTA plasma		EDTA plasma		EDTA plasma		EDTA plasma		EDTA Plasma /Serum	
*Platform*	*Simoa*	Simoa HD-X		Simoa HD-X		Simoa HD-X		Simoa HD-X		Simoa HD-X		Simoa HD-x/HD-1	
*Antibodies*	*Name capture*	AT270		ADx252		AT270		FAb2 of IBA493		ADx253		ADx253	
	*Epitope capture (AA) according to tau 441 numbering*	Sequence 176-PPAPKT(p)P-182 phosphorylated specifically at threonine-181		Phospho-Thr 181 and no cross-reactivity with phospho-Thr175		Sequence 176-PPAPKT(p)P-182 phosphorylated specifically at threonine-181		Peptide phosphorylated at Thr217		Phosphorylated tau at T231		Phosphorylated tau at T231	
	*Name detector*	LRL		ADx204		Tau12		4G10E2		ADx204		Tau12	
	*Epitope detector (AA)*	111–130 according to the Tau441 sequence		N-terminal, that recognizes all forms of tau except those phosphorylated at Tyr 18		N-terminal epitope 6-QEFEVMEDHAGT-18		111–130 according to the Tau441 sequence		N-terminal, that recognizes all forms of tau except those phosphorylated at Tyr 18		N-terminal epitope 6-QEFEVMEDHAGT-18	
*Assay protocol*	*Steps*	2-step assay		2-step assay		2-step assay		3-step assay		2-step assay		3-step assay	
	*Incubation times, min*	60-5		60-10.5		35-5		30-5.15-5.15		60-5.15		40-7-7	
	*Sample/calibrator volume, μL*	100		135		100		100		100		100	
	*Beads volume, μL*	25		25		25		25		25		25	
	*Detector volume, μL*	20		20		20		100		20		100	
	*SBG volume, μL*	100		100		100		100		100		100	
*Assay reagents*	*Helper beads, % of beads*	66%		50%		60%		50%		50%		0%	
	*SBG, pM*	150 pM		50pM		150 pM		150 pM		50 pM		300	
	*Detector, μg/mL*	1		0.6		*Unknown*		0.1		0.6		2	
*Calibrator*	*Type*	Synthetic peptide		Synthetic peptide		Recombinant protein		Synthetic peptide		Synthetic peptide		Recombinant protein	
	*No. of calibrator points*	9		8		7		8		8		8	
	*Range, pg/mL*	0.226-52		0.625-50		0.177-86		0.04-180		0.3125-40		0-64	
	*Curve fit*	1/y^2^-weighted 5PL		1/y^2^-weighted 5PL		1/y^2^-weighted 4PL		1/y^2^-weighted 4PL		1/y^2^-weighted 5PL		1/y^2^-weighted 4PL	
*Sample dilution*	*Fold-dilution*	4		5		4		2		5		2	
	*Recommended method*	Automated		Manual		Automated		Automated		Manual		Automated	
**Assay sensitivity and precision results**													
*Sensitivity*	*Analytical LLOQ, pg/mL*	1.55		2.36		0.24		0.15		0.56		3.95	
	*Functional LLOQ, pg/mL*	6.2		11.8		0.96		0.3		2.8		7.9	
*Concentrations of QC and KC panels*	*QC1: high, pg/mL*	15		26.8		3.8		2		7.4		26.7	
	*QC2: intermediate, pg/mL*	6.9		13.3		1.3		0.6		4.7		17.1	
	*QC3: low, pg/mL*	5.9		9.6		1.1		0.2		2.6		8.3	
	*KC1, pg/mL*	4.05		9.75		3.22		0.64		1.41		*NA*	
	*KC2, pg/mL*	16.69		17.87		70.35		1.99		5.72		*NA*	
	*KC3, pg/mL*	142.01		*N/A*		*N/A*		61.6		*N/A*		*NA*	
*Precision QCs*	*Average Intra-assay %CV*	6.6		14.5		7.7		13.5		16.8		3.7	
	*Average Inter-assay %CV*	10		15.2		19.5		14.1		27.7		5.1	
*Precision KCs*	*Average Intra-assay %CV*	5.6		16		6		9		19.9		*NA*	
	*Average Inter-assay %CV*	9		23		29.5		10.5		30.5		*NA*	
*Clinical samples measurements*	*Number*	80		80		80		80		80		80	
	*Range concentration, pg/mL*	2.69–21.65		1.91–77.29		0.89–8.65		0.04–1.93		1–16.07		5.68–25.8	
	*Within calibrator range, %*	100%		100%		100%		100%		100%		100%	
	*Range, CV%*	0.00–23.23		0.33–69.08		0.1–15.91		0.07–64		0.05–51.34		0.01–14.49	
	*Average CV%*	5.74		12.20		5.83		14.20		8.25		3.35	
	*n measured <LLOQ*	21		14		1		39		1		7	
	*n measured > 20%CV*	1		13		0		16		3		0	
**Other validation results**													
*Parallelism*	*Average slope of samples*	0.67		0.43		0.49		0.59		0.61		0.90	
	*Range of slopes of samples*	0.55–0.75		0.33–0.61		0.39–0.61		0.53–0.68		0.48–0.72		0.85–0.97	
	*Average slope of calibrator*	0.67		0.39		0.52		0.60		0.72		0.78	
	*Parallelism, %*	99.6		110.3		94.6		98.7		84		116	
*Dilution linearity*	*Spiked concentration, pg/ml*	150		150		150		11		150			
		Df (x)	Mean %L	Df (x)	Mean %L	Df (x)	Mean %L	Df (x)	Mean %L	Df (x)	Mean %L	Df (x)	Df (x)
	*Linear dilution factor with mean %Linearity*	1	-	1	-	1	-	1.00	-	1	-	1	1
		5	173	5	54	5	200	2.80	118	5	147	5	5
		25	132	25	139	25	117	7.84	125	25	81	25	25
		125	120	125	117	125	118	21.95	115	125	112	125	125
		625	294	625	376	625	153	61.47	109	625	150	625	625
		3125	426	3125	541	3125	265	172.10	134	3125	196	3125	3125
*Recovery*		Spike	Mean %R	Spike	Mean %R	Spike	Mean %R	Spike	Mean %R	Spike	Mean %R	Spike	Mean %R
	*Spiked concentration (pg/mL)* *With mean %Recovery*	0.8	102	0.85	54	0.8	72	0.4	108	0.85	131	1	150
		4.0	95	4.27	67	4.0	82	1	107	4.27	139	6	124
		20.0	149	21.33	67	20.0	83	4	113	21.33	147	24	113

Phospho-specific antibody FAb2 of IBA493 and anti-tau antibodies LRL and 4G10E2 are property of Eli Lilly and Company. Phospho-specific antibody ADx252, ADx253, and anti-tau antibody ADx204 are property of ADx NeuroSciences. Phospho-specific AT270 is of ThermoFischer Scientific, and Tau-specific Tau12 is of Sigma Aldrich. Analytical LLOQ was calculated as the mean signal of 16 blanks plus 10 times the SD, with the P-tau concentration extrapolated from the calibration curve. This was multiplied by the sample dilution factor to obtain the functional LLOQ. QC samples are EDTA plasma pools and specific to each assay. KC samples were from the providers and specific to each assay, either synthetic peptide or recombinant protein spiked in buffer (both Eli Lilly assays and P-tau181 Quanterix assay, respectively) or remnant EDTA plasma sample (both ADx assays). KCs were not available for the P-tau231 Gothenburg assay. Average intra-and inter-assay variation was derived from measuring the QC and KC panels over four independent runs (except only two runs for the high QC sample with the P-tau181 ADx). With each assay, 80 clinical samples were measured, but due to technical reasons duplicate results were obtained, for 66 samples with P-tau181 Eli Lilly, for 74 with P-tau181 ADx, for 79 with P-tau217 Eli Lilly, for 79 with P-tau231 ADx and for 74 with P-tau231 Gothenburg. No results were obtained for 3 samples with P-tau181 Eli Lilly, for 1 sample with P-tau181 ADx and for 3 samples with P-tau231 Gothenburg. For parallelism, with each assay, four samples were measured after being four-times 2-fold serially diluted (P-tau181 Eli Lilly, P-tau181 ADx, and P-tau231 ADx: starting dilution 5-fold, reaching 40-fold; P-tau181 Quanterix: starting dilution 4-fold, reaching 32-fold; P-tau217 Eli Lilly and P-tau231 Gothenburg: starting dilution 2-fold, reaching 16-fold). For dilution linearity, three samples were spiked with high recombinant protein concentration, subsequently measured undiluted, and serially diluted until low P-tau concentrations below the LLOQs of the assays. With the P-tau181 ADx assay, two out of three of the undiluted samples were not measurable, likely due to matrix effect. With the P-tau231 Gothenburg, signals were not detected for the lowest two dilutions with the three samples

*P-tau* Phosphorylated tau, *SBG* Streptavidin β-galactosidase, *PL* Polynomial, *LLOQ* Lower limit of quantification, *QC* Quality control, *KC* Kit control, *CV* Coefficient of variation, *%L* % linearity, *%R* % recovery, *NA* Not applicable

All assays use different capture and detector antibodies or different combinations of them. All assays were calibrated with seven to nine calibrator points using a synthetic peptide, except the P-tau181 Quanterix assay that used a recombinant protein (full-length recombinant tau1-441 phosphorylated in vitro by glycogen synthase kinase 3β; TO8–50FN; SignalChem, Vancouver, BC, Canada) [5]. For the Eli Lilly assays (P-tau181 and P-tau217), two different synthetic peptides were used. For both ADx assays (P-tau181 and P-tau231), one single synthetic peptide with a phosphorylation on both threonine 181 and 231 was used. For P-tau231 Gothenburg, full-length recombinant tau 441 phosphorylated in vitro by glycogen synthase kinase 3β was used as the calibrator.

### Analytical validation of the P-tau assays

We validated the sensitivity (lower limit of quantification; LLOQ), precision, parallelism, dilution linearity, and recovery of all P-tau assays according to the method developed by the BIOMARKAPD consortium [23] (details in Table 1).

For each assay, LLOQ was calculated from the mean signal of 16 blanks plus 10x SD, with the P-tau concentration interpolated from the calibration curve (i.e., the analytical LLOQ) and subsequently multiplied by the sample dilution factor (i.e., the functional LLOQ). Intra-assay precision was derived from the duplicate measurements of the 80 clinical samples. Inter-assay precision was calculated by measuring assay-specific quality control (QC) panels of three pooled EDTA plasma samples (high, medium, and low) and two or three kit controls (provided by the manufacturers; except for the P-tau231 Gothenburg assay that did not include KCs) over four runs (except for QC high with P-tau181 ADx, which was measured twice). Parallelism was calculated as the average %-agreement of the slope of the assay calibrator with the slopes of four and 4-times serially diluted plasma samples. For P-tau181 Eli Lilly, P-tau181 ADx, and P-tau231 ADx, the starting dilution was 5-fold, with a subsequent 2-fold serial dilution until 40-fold. For P-tau181 Quanterix, the starting dilution was 4-fold with a subsequent 2-fold dilution until 32-fold. For P-tau217 Eli Lilly and P-tau231 Gothenburg assays, the starting dilution was 2-fold, with a subsequent 2-fold serial dilution until 16-fold. Parallelism results measured below the LLOQ were not excluded from the calculations. Dilution linearity was assessed using three samples with an assay-specific spike that were measured undiluted and serially diluted until ultimately low levels below LLOQ were reached. The average %-agreement of P-tau concentration in a serially diluted sample was calculated in comparison to the P-tau concentration measured in the previous dilution. Recovery of each assay was determined by measuring P-tau in four plasma samples that were diluted according to their standard dilution factor and spiked with a low, medium, and high spike of the assay-specific calibrator, in comparison to non-spiked sample.

### Data analysis

Acceptance criteria were < 20% coefficient of variation (CV) for precision, or between 80 and 120% for %-agreement calculations. For the clinical results, we used Spearman’s correlations to assess the agreement of P-tau measurements between the assays. We used non-parametric Mann-Whitney *U* tests to compare P-tau levels between the AD dementia and control groups. Since we included 6 assays in this study for P-tau measurement, the *p* value that we considered significant for these group comparisons after Bonferroni correction was *p* < 0.05/6 = *p* < 0.0083. We applied receiver operating characteristic curve (ROC) analysis to calculate the accuracy of the P-tau assays to discriminate between AD dementia and controls and calculated cutoffs at the Youden’s indices [24]. We applied repeated measures ANOVA using natural log-transformed and *Z*-transformed P-tau levels as within-subject variables and group (AD dementia versus control) as between-subject variables, to investigate if the assays have a different discriminatory potential. As a post hoc analysis, we explored the difference in discriminatory accuracy of the assays by comparing their area under the curves (AUC) obtained with the ROC analyses using the DeLong test [25], in which we regarded *p* < 0.05 as significant and *p* < 0.10 as a trend. For all P-tau measurements below LLOQ, we used interpolated concentration as assigned by the Simoa. As a sensitivity analysis, we re-ran our analyses in the set with complete P-tau data for all assays (*n* = 37 controls, *n* = 36 patients with AD dementia), which did not change the findings (results not shown). We used R version 4.0.3 and SPSS version 26 for statistical analysis and construction of graphs.

## Results

### Analytical performance of the P-tau assays

#### LLOQ and precision

**Fig. 1:**
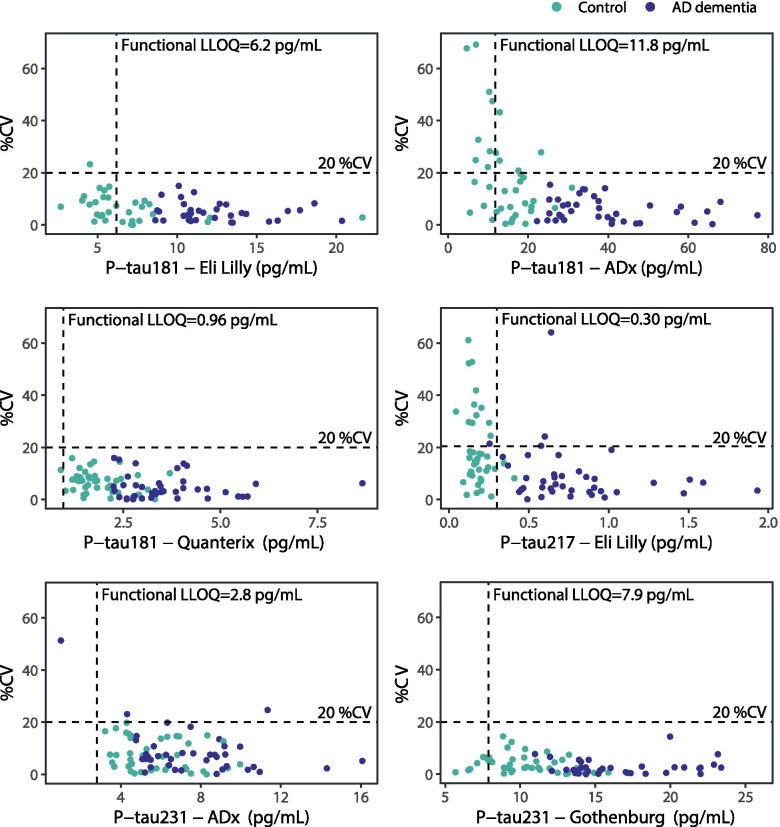
Precision plots of the six P-tau assays. For each assay, concentrations were plotted against the variation in their duplicate measurements (%CV), color-coded for clinical group. Due to technical reasons, duplicate results were obtained for 66/80 samples with P-tau181 Eli Lilly, for 74/80 with P-tau181 ADx, for 79/80 with P-tau217 Eli Lilly, for 79/80 with P-tau231 ADx, and for 77/80 with P-tau231 Gothenburg. Horizontal dashed lines were set at CV 20%, vertical dashed lines were set at the functional LLOQ for each assay (i.e., analytical LLOQ multiplied by sample dilution factor). CV coefficient of variation; P-tau phosphorylated tau, LLOQ lower limit of quantification

Functional LLOQ and intra-assay precision results are presented in Table 1 and Fig. 1. With P-tau181 Eli Lilly, 21 samples (27%; controls) were measured below the functional LLOQ and one sample (1%) was measured with a %CV > 20%. For P-tau181 ADx, levels of 14 samples (18%) were below LLOQ and 13 samples (18%) had a %CV > 20%. For P-tau181 Quanterix, level of only one sample (1%) was below LLOQ, and none of the samples had a %CV > 20%. For P-tau217 Eli Lilly, levels of 39 samples (49%; 37 of which were controls) were below LLOQ and 16 samples (20%) had a %CV > 20%. For P-tau231 ADx, level of 1 sample (1%) was below LLOQ and 3 samples (4%) had a %CV > 20%. For P-tau231 Gothenburg, 7 samples (9%) were measured below LLOQ and none of the samples had a %CV > 20%.

For both the in-house plasma quality controls (QC) and the manufacturer-provided kit controls (KC), average inter-assay precision was acceptable for all assays (Table 1), ranging from 5.1 (P-tau231 Gothenburg) to 27.7% (P-tau231 ADx) for the QCs (individual values in supplementary table 1) and from 9 (P-tau181 Eli Lilly) to 30.5% (P-tau231 ADx) for the KCs (individual values in supplementary table 2).

#### Parallelism, dilution linearity, and recovery

**Fig. 2:**
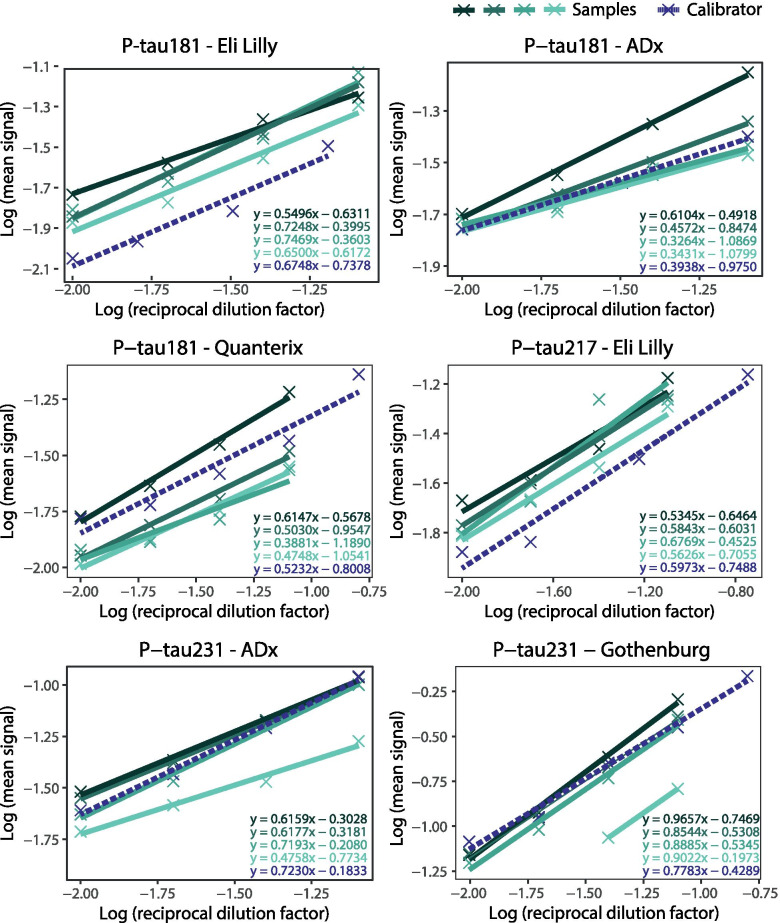
Parallelism of the six P-tau assays. Serial dilution of four plasma samples (in green, solid lines) and one calibrator (in purple, dashed line) was performed for each of the P-tau assays. Plasma samples with relatively high endogenous P-tau concentrations were selected for the parallelism experiment. For P-tau231 Gothenburg, one sample showed no signals upon dilution for the 8- and 16-fold dilutions. Crosses represent the individual measurements. A linear slope was fitted for each sample and for the calibrator, the equation of which is presented in the figures

All P-tau assays showed good parallelism (Table 1, Fig. 2), with average parallelism ranging from 84% (P-tau231 ADx) to 116% (P-tau231 Gothenburg).

Dilution linearity responses of the spiked samples upon serial dilution varied between the P-tau assays (Table 1). The P-tau217 Eli Lilly assay showed the broadest dilution range (i.e., linear responses from undiluted until 61.5-fold diluted), followed by P-tau181 Quanterix and P-tau231 ADx (5-fold until 125-fold diluted). The dilution range with acceptable linearity was narrowest for P-tau181 Eli Lilly (25-fold until 125-fold), P-tau181 ADx (25-fold until 125-fold), and P-tau231 Gothenburg (5-fold until 25-fold). No hook effect at spiked concentrations above the highest calibrator point of the assays was observed for any of the assays.

Average %-recovery varied between the assays (Table 1). For P-tau217 Eli Lilly, all spikes (low, intermediate, and high) showed acceptable recovery. For P-tau181 Eli Lilly, the low and intermediate spikes showed acceptable recovery. For P-tau181 Quanterix, the intermediate and high spikes showed acceptable recovery. For P-tau181 ADx, P-tau231 ADx, and P-tau231 Gothenburg, none of the low, intermediate, or high spikes showed acceptable recovery.

### Clinical performance of the P-tau assays

**Fig. 3:**
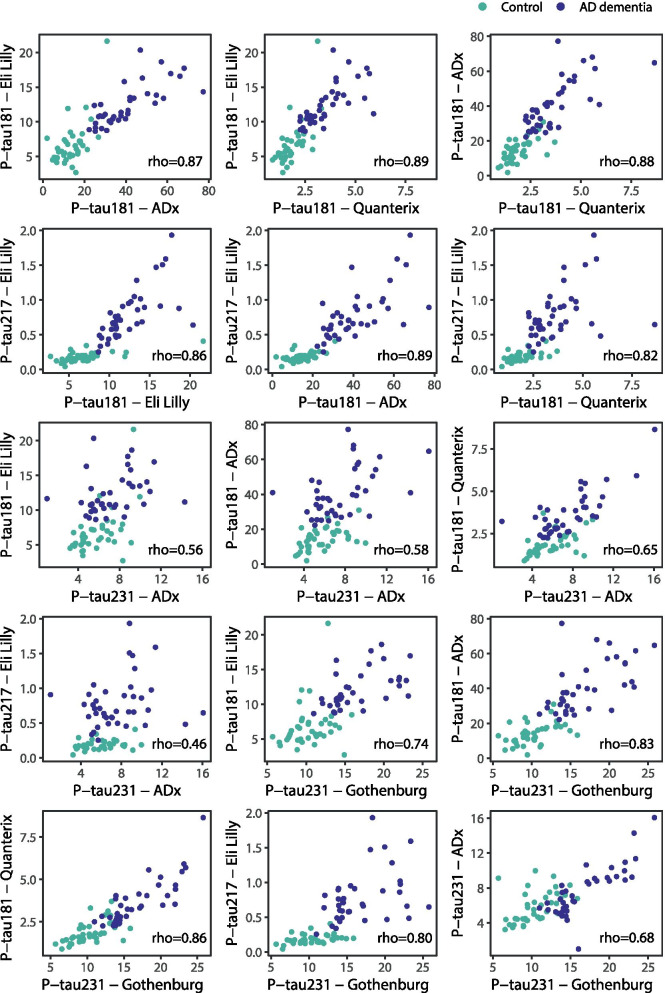
Scatterplots of the six P-tau isoforms, color-coded for diagnostic group. Correlation coefficient rho is calculated using Spearman’s rank correlation. Controls are presented in green and patients with AD dementia in purple. P-tau phosphorylated tau

The P-tau levels measured in the plasma samples of 40 controls and 40 patients with AD dementia with the different assays correlated moderately to strongly with each other (Fig. 3; all *p* < 0.001)). Strong correlations were observed among the results obtained with the P-tau181 assays (range Spearman’s rho 0.87–0.89), for P-tau217 Eli Lilly results with results of all three P-tau181 assays (range 0.82–0.89) and with results of the P-tau231 Gothenburg assay (rho 0.80) and for P-tau231 Gothenburg results with results of all three P-tau181 assays (range 0.74–0.86). Correlations for P-tau231 ADx results with results of all other assays were moderate (range 0.46–0.68).

**Fig. 4:**
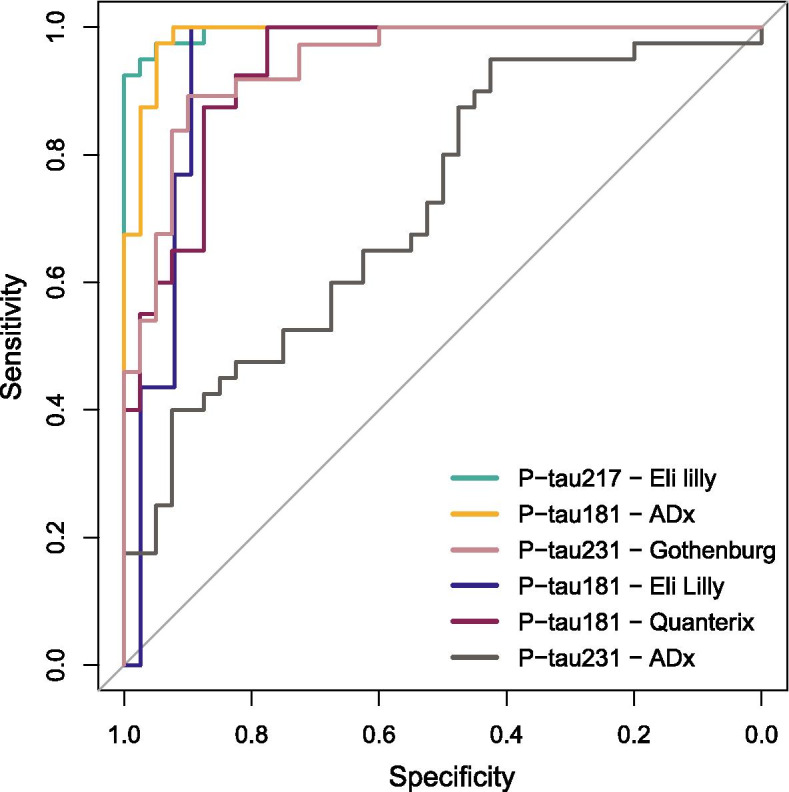
ROC curves discriminating between controls and AD dementia for the six P-tau assays. P-tau: phosphorylated tau

With all assays, P-tau levels were significantly higher in AD dementia patients compared to controls (all: *p* < 0.001; Table 2; supplementary fig. 1). For P-tau217 Eli Lilly, median levels were 4.1-fold higher in AD dementia compared to controls, compared with 1.8-fold for P-tau181 Eli Lilly, 2.9-fold for P-tau181 ADx, 1.9-fold for P-tau181 Quanterix, 1.3-fold for P-tau231 ADx, and 1.5-fold for P-tau231 Gothenburg. AUCs for differentiating controls and patients with AD dementia ranged from 0.936 to 0.995 for P-tau217 Eli Lilly, all P-tau181 assays and P-tau231 Gothenburg assay, and was AUC = 0.719 for P-tau231 ADx (Table 2, Fig. 4). Repeated measures ANOVA demonstrated that there were differences between the P-tau assays in their discriminatory potential (interaction P-tau assay*group: Wilks’ Lambda *p* < 0.001). Post hoc comparison of the AUCs of the ROC analysis showed that P-tau181 ADx and P-tau217 Eli Lilly performed comparably good (DeLong’s *p* = 0.38), while both assays outperformed P-tau181 Quanterix (P-tau181 ADx: ΔAUC = 0.05, DeLong’s *p* = 0.03; P-tau217 Eli Lilly: ΔAUC = 0.06, DeLong’s *p* = 0.02). P-tau217 Eli Lilly outperformed P-tau231 Gothenburg (ΔAUC = 0.05, DeLong’s *p* = 0.03) and P-tau181 ADx tended to outperform P-tau231 Gothenburg (ΔAUC = 0.04, DeLong’s *p* = 0.08). Furthermore, both P-tau217 Eli Lilly and P-tau181 ADx tended to perform better than P-tau181 Eli Lilly did (P-tau181 ADx: ΔAUC = 0.05, DeLong’s *p* = 0.08; P-tau217 Eli Lilly: ΔAUC = 0.06, DeLong’s *p* = 0.07). The P-tau231 ADx assay was outperformed by all assays (range ΔAUC 0.22–0.28; all *p* < 0.001).

**Table 2:** Clinical performance of the six P-tau assays

	AD dementia	Controls	Differentiation AD dementia versus controls				
	Median [IQR]	Median [IQR]	Fold change	AUC (95% CI)	Cutoff	%Sens	%Spec
*P-tau181 Eli Lilly*	11.1 [10.4–13.6]	6.1 [5.1–7.4]	1.8	0.938 (0.872–1.000)	8.6	100	89
*P-tau181 ADx*	37.6 [28.8–48.6]	13.2 [10.3–17.6]	2.9	0.988 (0.969–1.000)	24	100	92
*P-tau181 Quanterix*	3.4 [2.7–4.1]	1.6 [1.4–2.2]	2.0	0.936 (0.885–0.987)	2.2	100	78
*P-tau217 Eli Lilly*	0.7 [0.6–0.9]	0.17 [0.14–0.2]	4.1	0.995 (0.987–1.000)	0.42	92.5	100
*P-tau231 ADx*	7.3 [5.6–9.1]	5.5 [4.5–6.9]	1.3	0.719 (0.607–0.831)	4.7	95	43
*P-tau231 Gothenburg*	15.3 [13.9–19.8]	10.3 [8.9–11.9]	1.5	0.943 (0.896–0.991)	13.4	89	90

Median concentrations are in pg/mL. Fold change was calculated by dividing the median concentration in the AD dementia group over the median concentration in the control group. AUCs were derived from ROC analysis. All group comparisons were significant with p values below the Bonferroni-adjusted p value cutoff of 0.0083. P-tau cutoff was specified at the Youden’s indeces (maximum sum of sensitivity and specificity)

*P-tau* Phosphorylated tau, *AD* Alzheimer’s disease, *IQR:* Interquantile range, *AUC* Area under the curve, *sens* Sensitivity, *spec* Specificity

## Discussion

We here compared six assays of four providers that detect plasma P-tau isoforms 181, 217, and 231 on the Simoa, of which five are prototype assays and one is currently commercially available. With all assays, P-tau concentrations were measured in all clinical samples above the assay blanks, but for some assays particularly (P-tau181 Eli Lilly, P-tau181 ADx, and P-tau217 Eli Lilly), a large proportion of control samples were measured below the LLOQs of the assays. Furthermore, all assays showed good analytical performance, especially in terms of intra- and inter-assay precision and parallelism. Clinically, we found that P-tau levels with all assays were higher in patients with AD dementia compared to controls and that particularly P-tau217 Eli Lilly assay, all three P-tau181 assays and the P-tau231 Gothenburg assay demonstrated high diagnostic accuracy for AD dementia (AUC > 0.93). The prototype P-tau231 ADx assay showed moderate diagnostic accuracy for AD dementia (AUC = 0.72). Our extensive and systematic assay comparison in terms of analytical characteristics combined with a clinical validation in a small proof-of-concept cohort are relevant for the interpretation of results that are currently being obtained across cohorts and studies using different forms or versions of the available P-tau assays.

Precise quantification of P-tau in all samples, including control samples, is relevant to obtain reliable results, e.g., to detect slight increases in early disease stages. With all six Simoa P-tau assays, we obtained detectable P-tau levels above the assay blanks in all clinical samples, including the samples of the healthy controls. However, especially with the P-tau217 Eli Lilly and P-tau181 ADx assays, many control samples were measured below LLOQ, resulting in higher imprecision (i.e., CV > 20%). This could impose difficulties on the clinical use of the two assays as stand-alone diagnostic tests, especially in early AD stages. By contrast, the P-tau181 Eli Lilly, P-tau181 Quanterix, P-tau231 ADx, and P-tau231 Gothenburg assays showed more robust intra-assay precision profiles. Although with the P-tau181 Eli Lilly assay, almost half of the control samples were measured below LLOQ, and with the P-tau231 Gothenburg 7 samples were measured below LLOQ, none of the measured samples had a CV% > 20%. With both the P-tau181 Quanterix and the P-tau231 ADx assays, only one sample was measured below LLOQ and respectively only one and three samples had a CV > 20%. In agreement with the intra-assay precision plots, particularly QC plasma samples with low concentrations close to or below the LLOQs of the assays showed variability of the results over the independent runs. Similar high variability was also noted for kit controls with concentrations close to or below the LLOQs. For other purposes, we used the P-tau181 Quanterix assay over 61 Simoa runs, using one assay lot, to monitor inter-assay precision over 3 months. We observed reproducible measurements with an average of 9% CV for the plasma QCs over time (data not shown). This extensive inter-assay precision data for P-tau181 Quanterix extends on the findings of our current study confirming that the P-tau181 Quanterix assay is a stable assay.

Analytically, the average parallelism responses for all assays were within the acceptable ranges, indicating good parallelism. Generally, parallelism indicates whether binding abilities of antibodies to endogenous P-tau isoforms is similar to the recombinant P-tau or synthetic P-tau peptide that is used as calibrator in the assays. For the dilution linearity, all P-tau assays showed acceptable dose-response linearity above their LLOQs. Particularly, P-tau217 Eli Lilly showed the broadest dilution linearity range. The range was slightly narrower for P-tau181 Quanterix and P-tau231 ADx and was narrowest for P-tau181 Eli Lilly, P-tau181 ADx, and P-tau231 Gothenburg. Narrow dilution ranges indicate that plasma matrix *and/or* sample diluent affect the detectability of P-tau isoforms differently; thus, sample dilution might affect accuracy and precision. The spike recoveries of the low, intermediate, and high spikes were all within the acceptable ranges for the P-tau217 Eli Lilly assay, while the recovery of one or more spikes deviated with the other assays. Deviations in recovery responses might suggest that the assay responds differently to endogenous and recombinant protein or synthetic peptide or that there is interference from biological samples leading to less accuracy in detecting true concentration differences between samples. Taking all results of the analytical performance of the assays together, P-tau181 Quanterix might be the favored assay, since precision profiles were robust, along with good parallelism, dilution linearity, and recovery. However, it is to note that all five other assays showed good analytical performance, and the prototype status of the assays should be considered as well. Especially, the P-tau217 had robust analytical performance (parallelism, linearity, recovery); however, the precision profiles on the clinical sample measurements showed less accuracy for measurements in low concentration samples (i.e., the controls).

Clinically, all P-tau181, P-tau217, and P-tau231 Gothenburg results correlated strongly with each other, while P-tau231 ADx correlated only moderately with the other results. The three P-tau181 assays and P-tau217 Eli Lilly and P-tau231 Gothenburg assays performed excellent in discriminating between controls and patients with AD dementia, with AUCs > 0.93, while the prototype P-tau231 ADx assay performed moderately with AUC = 0.72. The high AUCs observed for discriminating patients with AD dementia and controls with plasma P-tau181, P-tau217 Eli Lilly, and P-tau231 Gothenburg assays are in line with several previous reports in large cohorts using the same reagents but employing the MSD platform [4, 8, 16, 18, 26–28]. Likewise, the high AUC obtained with the P-tau181 Quanterix assay is in line with recent publications using a homebrew assay that employs the same antibodies as the Quanterix assay [5, 9]. Interestingly, in our study, the prototype P-tau231 ADx only moderately associated with AD, which contradicts the findings presented in this paper with the P-tau231 Gothenburg assay, or findings published earlier [11]. The assay set-up of the P-tau231 ADx is not exactly the same as the P-tau231 Gothenburg assay set-up, with a different antibody used as the detector and differences in terms of diluents used and assay conditions, such as reagent incubation times. It remains to be investigated if this difference in detector antibody or the differences in the assay set-ups explain the difference in clinical findings between the assays.

Tau proteins are generated from the same gene, and subsequently subjected to post-translational modifications, such as phosphorylation at different sites, producing different forms (e.g., P-tau217, P-tau181, and P-tau231). Some post-translational modifications were reported to occur at earlier stages of the AD disease process in the brain tissue [29]. Therefore, it was suggested that different P-tau forms might be favorable in different contexts of use, for example specifically to investigate ongoing AD pathology in cognitively unimpaired individuals or to discriminate patients with AD dementia from patients with other types of dementia. Recently, using a mass spectrometry approach that simultaneously quantifies different P-tau forms, the different phosphorylation sites that are quantified in this study were not phosphorylated to the same degree, with phosphorylation of P-tau181 present to a high degree in plasma and CSF of controls and with a relative small increase in patients with AD, while P-tau217 seems to be phosphorylated to a very low degree in controls, while the increase in AD is high [6, 17]. This suggests that P-tau217 might be a more sensitive marker than P-tau181 for AD pathological changes. However, our data did not support this, since AUCs for P-tau181 ADx were similar to P-tau217 Eli Lilly. A recent study [11] reported that plasma P-tau231 Gothenburg had similar clinical performance as the P-tau181 Simoa assay [5], similar to the findings or our current work. From our study, it cannot be excluded that technical differences between P-tau assays, such as antibodies and binding specificity may underlie subtle differences observed in large clinical comparison studies. In addition to analytical robustness and clinical validity, there are also other factors to consider, such as widespread availability for research and clinical settings, which usually occurs through commercialization. At this point, only the P-tau181 Quanterix is commercially available as a research assay. The five prototype assays compared in this study showed promising results in terms of analytical and clinical validity; thus, their further development into commercial products will be important, to make these assays available to the wide research community. Improvement of assay sensitivity to increase measurement precision in the low concentration ranges will be important for the assays with many control samples measured below the LLOQ, and buffer adjustments to decrease matrix effects is needed to improve recovery results.

A particular strength of our study is the extensive systematic analytical validation in EDTA plasma samples in one clinical research laboratory, allowing to directly compare the performance of the six novel ultrasensitive Simoa P-tau assays. Extensive comparison of clinical contexts of use of different P-tau assays is subject of several ongoing efforts, but none compared the analytical robustness yet.

### Limitations

Regarding the limitations, we did not include all possible assays that arise in this quickly emerging field. For example, we did not include the in-house set-up of the P-tau181 assay as described by Karikari et al. [5], due to suboptimal technical performance of this assay in our lab during pilot testing. However, it is noted that this same pair of antibodies is used in the commercial P-tau181 Quanterix assay. Moreover, we did not compare the Simoa and MSD set-ups for the Eli Lilly assays. Therefore, we cannot conclude if Simoa had added value in terms of increasing assay sensitivity of the MSD assay set-ups. In addition, studies assessing the commutability of the assays, using reference materials for the harmonization of plasma P-tau measurements, will further improve our understanding of potential differences between plasma P-tau assays for clinical use over the Alzheimer’s continuum. Furthermore, the clinical sample set was small and exploratory, limiting the conclusions pertaining to different possible clinical contexts of use. Finally, our controls were not confirmed to be negative for ongoing amyloid pathology as no brain amyloid PET or CSF amyloid-beta42 data was available for these participants. However, since all samples were measured with all assays, this should not affect our comparative analyses.

## Conclusions

In conclusion, all investigated assays showed robust analytical performance, and all yielded accurate clinical discrimination, except the current version of P-tau231 ADx assay. Differences in the analytical performance of the six P-tau assays may underlie subtle differences observed in clinical comparison studies, including in our current work.

## Supplementary Information

**Additional file 1: Supplementary figure 1.** P-tau isoforms measured with different assays in AD dementia and control samples. **Supplementary table 1.** Intra-and Inter-assay %CV for the P-tau assays using QC samples. **Supplementary table 2.** Intra-and Inter-assay %CV for the P-tau assays using kit controls provided by the manufacturers of the assays.

## Data Availability

The datasets generated and analyzed during the current study can be made available by the corresponding author upon reasonable request.

## Abbreviations

AD: Alzheimer’s disease
Aβ: Amyloid-β
P-tau: Phosphorylated tau
PET: Positron emission tomography
CSF: Cerebrospinal fluid
P-tau181: Phosphorylated tau at threonine 181
P-tau217: Phosphorylated tau at threonine 217
P-tau231: Phosphorylated tau at threonine 231
K2EDTA: Potassium Ethylenediaminetetraacetic acid
NIA-AA: National Institute on Aging and Alzheimer’s Association
LLOQ: Lower limit of quantification
BIOMARKAPD: Biomarkers for Alzheimer’s disease and Parkinson’s disease
QC: Quality control
CV: Coefficient of variation
ROC: Receiver operating characteristic curve
AUC: Area under the curves
ANOVA: Analysis of variance
