# Supplementary material for: Clinical and analytical comparison of six Simoa assays for plasma P-tau isoforms P-tau181, P-tau217, and P-tau231
Source: Alzheimers Res Ther. 2021 Dec 4;13:198. doi: 10.1186/s13195-021-00939-9 (PMC8645090; doi:10.1186/s13195-021-00939-9)
Supplement: Supplementary file 1 — Additional file 1: Supplementary figure 1. P-tau isoforms measured with different assays in AD dementia and control samples. Supplementary table 1. Intra-and Inter-assay %CV for the P-tau assays using QC samples. Supplementary table 2. Intra-and Inter-assay %CV for the P-tau assays using kit controls provided by the manufacturers of the assays. [file 13195_2021_939_MOESM1_ESM.docx]

**Clinical and analytical comparison of six Simoa assays for plasma P-tau isoforms P-tau181, P-tau217 and P-tau231**

Sherif Bayoumy, MSc^1*†^; Inge M. W. Verberk, MSc^1*^; Ben den Dulk, PhD^1^; Zulaiga Hussainali^1^; Marissa Zwan, PhD^2^; Wiesje M. van der Flier, PhD^2,3^; Nicholas J. Ashton, PhD^4,5,6,7^; Henrik Zetterberg, PhD^4,8,9,10,11^; Kaj Blennow, PhD^4,8^; Jeroen Vanbrabant, PhD^12^; Erik Stoops, MSc^12^; Eugeen Vanmechelen, PhD^12^; Jeffrey L. Dage^13,14^, Charlotte E. Teunissen, PhD^1^

^1^ Neurochemistry Laboratory, Department of Clinical Chemistry, Amsterdam Neuroscience, Vrije Universiteit Amsterdam, Amsterdam UMC, Boelelaan 1117, 1081 HV, Amsterdam, The Netherlands.

^2^ Alzheimer Center, Department of Neurology, Vrije Universiteit Amsterdam, Amsterdam UMC, Amsterdam, The Netherlands.

^3^ Department of Epidemiology and Data Science, Vrije Universiteit Amsterdam, Amsterdam UMC, Amsterdam, The Netherlands.

^4^ Department of Psychiatry and Neurochemistry, Institute of Neuroscience and Physiology, The Sahlgrenska Academy at the University of Gothenburg, Mölndal, Sweden

^5^ Wallenberg Centre for Molecular and Translational Medicine, University of Gothenburg, Gothenburg, Sweden

^6^ King’s College London, Institute of Psychiatry, Psychology and Neuroscience, Maurice Wohl Institute Clinical Neuroscience Institute, London, UK

^7^ NIHR Biomedical Research Centre for Mental Health and Biomedical Research Unit for Dementia at South London and Maudsley NHS Foundation, London, UK

^8^ Clinical Neurochemistry Laboratory, Sahlgrenska University Hospital, Mölndal, Sweden.

^9^ Department of Neurodegenerative Disease, UCL Institute of Neurology, Queen Square, London, UK.

^10^ UK Dementia Research Institute at UCL, London, UK.

^11^ Hong Kong Center for Neurodegenerative Diseases, Hong Kong, China.

^12^ ADx NeuroSciences NV, Technologiepark 94, Gent, Belgium.

^13^ Eli Lilly and Company, Indianapolis, IN 46285, USA.

^14^ Stark Neuroscience Research Institute, Indiana University School of Medicine, Indianapolis, IN 46202, USA.

* Shared first authorship; authors contributed equally.

† Corresponding author: Sherif Bayoumy. Neurochemistry Laboratory, Department of Clinical Chemistry, Amsterdam Neuroscience, Vrije Universiteit Amsterdam, Amsterdam UMC, Boelelaan 1117, 1081 HV, Amsterdam, The Netherlands. Telephone: +31204443029. Fax: +31204448529. Email: s.s.a.bayoumy@amsterdamumc.nl.

# SUPPLEMENTARY FIGURE


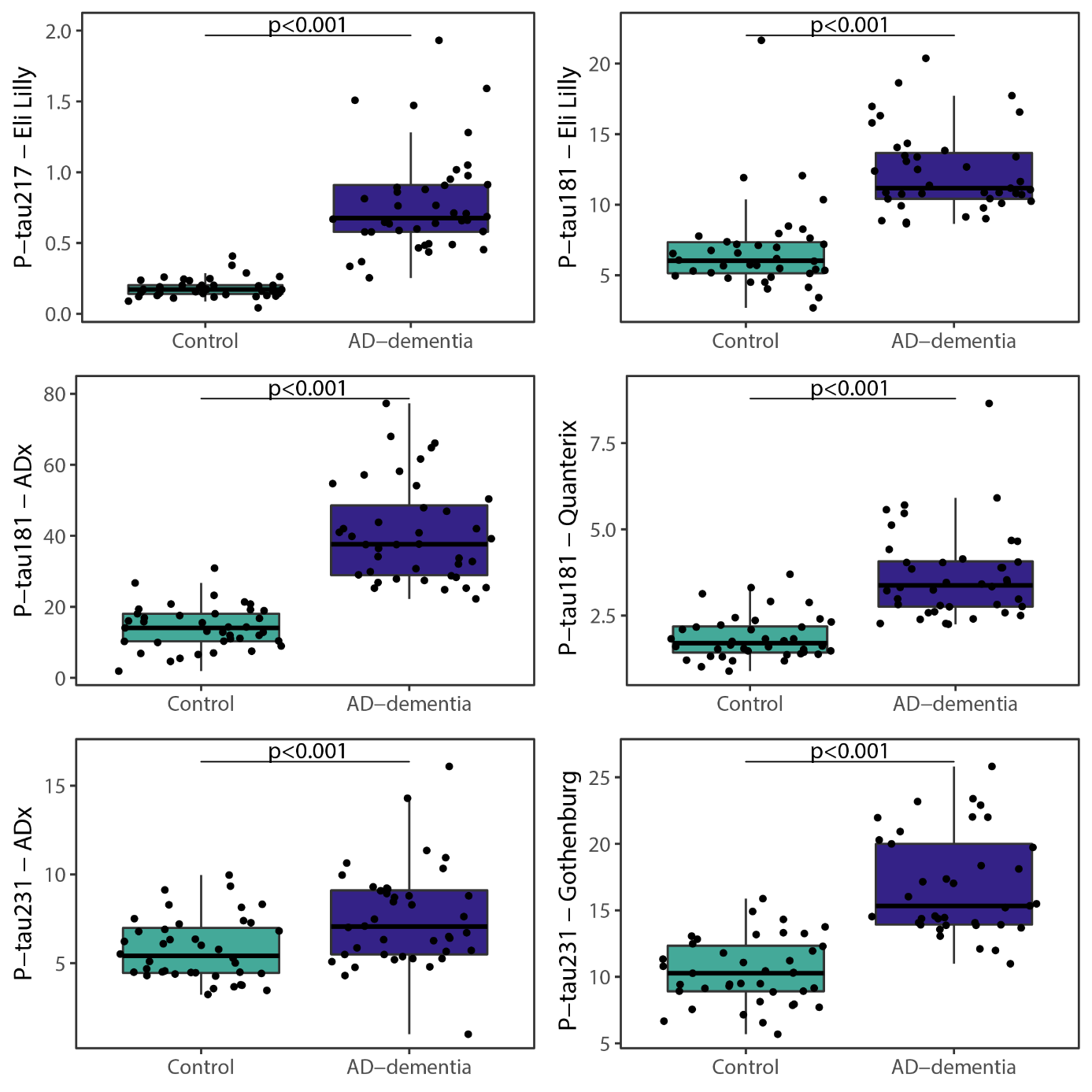


**Supplementary figure 1. P-tau isoforms measured with different assays in AD and control samples.** *p-values for group differences were calculated using non-parametric Mann-Whitney U Test. P-tau = phosphorylated tau, AD=Alzheimer’s Disease. All group comparisons were significant with p-values below the Bonferroni-adjusted p-value of 0.0083.*

# SUPPLEMENATRY TABLES

**Supplementary table 1.** Intra-and Inter-assay %CV for the P-tau assays using QC samples.

| Name Marker | Sample | Mean value | s_r_ | CV_r_/(%) | s_RW_ | CV_RW_/(%) |
| --- | --- | --- | --- | --- | --- | --- |
| P-tau 181 Eli Lilly | **HIGH** | 15,0 | 0,4 | 2,8 | 1,7 | 11,3 |
|  | **MEDIUM** | 6,9 | 0,8 | 10,9 | 0,8 | 11,6 |
|  | **LOW** | 5,9 | 0,3 | 6,0 | 0,4 | 7,0 |
| P-tau181 ADX | **HIGH** | 26,8 | 1,9 | 7,1 | 2,2 | 8,2 |
|  | **MEDIUM** | 13,3 | 1,6 | 12,2 | 1,8 | 13,4 |
|  | **LOW** | 9,6 | 2,3 | 24,0 | 2,3 | 24,0 |
| P-tau181 Quanterix | **HIGH** | 3,8 | 0,2 | 4,6 | 0,4 | 9,6 |
|  | **MEDIUM** | 1,3 | 0,1 | 6,1 | 0,1 | 10,9 |
|  | **LOW** | 1,1 | 0,1 | 12,5 | 0,4 | 38,0 |
| P-tau217 Eli Lilly | **HIGH** | 2,0 | 0,0 | 15,2 | 0,0 | 15,2 |
|  | **MEDIUM** | 0,6 | 0,1 | 15,8 | 0,1 | 16,8 |
|  | **LOW** | 0,2 | 0,2 | 9,5 | 0,2 | 10,4 |
| P-tau231 ADX | **HIGH** | 7,4 | 0,4 | 5,2 | 1,2 | 16,3 |
|  | **MEDIUM** | 4,7 | 0,7 | 14,4 | 1,0 | 22,1 |
|  | **LOW** | 2,6 | 0,8 | 30,7 | 1,2 | 44,6 |
| P-tau231 Gothenburg | **HIGH** | 26.7 | 0,32 | 3,9 | 0,3 | 4,1 |
|  | **MEDIUM** | 17.1 | 0,86 | 5,0 | 1,3 | 7,3 |
|  | **LOW** | 8.3 | 0,61 | 2,3 | 1,1 | 4,0 |

*r=repeatability; Rw=intermediate precision; CV=coefficient of variation.*

**Supplementary table 2**. Intra-and Inter-assay %CV for the P-tau assays using kit controls provided by the manufacturers of the assays.

| Name Marker | Sample | Mean value | s_r_ | CV_r_/(%) | s_RW_ | CV_RW_/(%) |
| --- | --- | --- | --- | --- | --- | --- |
| pTau181 Eli Lilly | **kit-control-1** | 4,0 | 0,3 | 8,4 | 0,6 | 15,2 |
|  | **kit-control-2** | 16,7 | 0,6 | 3,4 | 0,9 | 5,4 |
|  | **kit-control-3** | 142,0 | 7,2 | 5,1 | 9,7 | 6,8 |
| pTau181 ADX | **kit-control-1** | 17,9 | 2,4 | 13,3 | 3,3 | 18,4 |
|  | **kit-control-2** | 9,8 | 1,9 | 19,0 | 2,7 | 27,9 |
| pTau181 Quanterix | **kit-control-1** | 3,2 | 0,1 | 3,1 | 0,2 | 6,9 |
|  | **kit-control-2** | 70,4 | 6,7 | 9,5 | 36,6 | 52,0 |
| pTau217 Eli Lilly | **kit-control-1** | 0,6 | 0,1 | 9,9 | 0,1 | 10,0 |
|  | **kit-control-2** | 2,0 | 0,2 | 7,8 | 0,2 | 7,8 |
|  | **kit-control-3** | 61,6 | 5,7 | 9,2 | 8,4 | 13,7 |
| pTau231 ADX | **kit-control-1** | 1,4 | 0,4 | 25,4 | 0,6 | 43,7 |
|  | **kit-control-2** | 5,7 | 0,8 | 14,5 | 1,0 | 17,4 |
| P-tau231 Gothenburg | **kit-control-1** | NA | NA | NA | NA | NA |
|  | **kit-control-2** | NA | NA | NA | NA | NA |

*r=repeatability; rw=intermediate precision; CV=coefficient of variation; NA= not applicable.*
